# Obtaining Cellulose-Available Raw Materials by Pretreatment of Common Agro-Forestry Residues With *Pleurotus* spp.

**DOI:** 10.3389/fbioe.2021.720473

**Published:** 2021-09-22

**Authors:** Milica Galić, Mirjana Stajić, Jelena Vukojević, Jasmina Ćilerdžić

**Affiliations:** Faculty of Biology, University of Belgrade, Belgrade, Serbia

**Keywords:** delignification, laccase, lignocellulose, Mn-oxidizing peroxidases, white-rot fungi

## Abstract

The goals of the present study were to characterize the profile of ligninolytic enzymes in five *Pleurotus* species and determine their ability to delignify eight common agro-forestry residues. Generally, corn stalks were the optimal inducer of Mn-dependent peroxidase activity, but the activity peak was noted after wheat straw fermentation by *P. eryngii* (3066.92 U/L). *P*. *florida* was the best producer of versatile peroxidase, especially on wheat straw (3028.41 U/L), while apple sawdust induced the highest level of laccase activity in *P. ostreatus* (49601.82 U/L). Efficiency of the studied enzymes was expressed in terms of substrate dry matter loss, which was more substrate-than species-dependent. Reduction of substrate dry mass ranged between 24.83% in wheat straw and 8.83% in plum sawdust as a result of fermentation with *P*. *florida* and *P. pulmonarius*, respectively. The extent of delignification of the studied substrates was different, ranging from 51.97% after wheat straw fermentation by *P*. *pulmonarius* to 4.18% in grapevine sawdust fermented by *P. ostreatus*. *P*. *pulmonarius* was also characterized by the highest cellulose enrichment (6.54) and *P. ostreatus* by very low one (1.55). The tested biomass is a highly abundant but underutilized source of numerous value-added products, and a cocktail of ligninolytic enzymes of *Pleurotus* spp. could be useful for its environmentally and economically friendly transformation.

## Introduction

Lignocellulose accounts for about 60% of the total biomass on Earth and presents promising raw material for various industrial processes, such as production of bioethanol, paper, feed, food and numerous other value-added products (Stajić et al., 2009; Rastogi and Shrivastava, 2017). However, its complex chemical structure makes its utilization extremely demanding (Bilal et al., 2017). The most challenging phase in its transformation is removal of lignin, i.e., release of cellulose and hemicellulose for subsequent enzymatic hydrolysis. Lignin is a most recalcitrant natural compound, whose physical and chemical mineralization is neither ecologically nor economically justified. Therefore, the development of biological pretreatment systems represents the current trend in biotechnology (Meehnian et al., 2017). Owing to their well developed enzymatic system, fungi are a highly effective biofactory for lignocellulose conversion to cellulose-available resources. An enzyme cocktail composed of laccases, peroxidases and numerous auxiliary enzymes, makes white-rot mushrooms the most efficient delignifiers and potential participants in numerous biotechnological processes (Stajić et al., 2009; Knežević et al., 2014; Rudakiya and Gupte, 2017). However, despite numerous studies conducted during the past few decades, there is still a need for finding the most selective lignin remover, i.e., the species whose activity will retain the cellulose intact. It is well known that species of the genus *Pleurotus* are among the most efficient and selective mineralizators of lignin in agriculture residues (Stajić et al., 2004, 2006; Dong et al., 2013; Singh and Singh, 2014; Aditiya et al., 2016; Mustafa et al., 2016; Bilal et al., 2017; Fang et al., 2018; Matrínez-Patiño et al., 2018; Alfaro et al., 2020; de Souza et al., 2020). Their remarkable degradation ability is primarily based on the synthesis of numerous highly active laccase isoforms (Martínez et al., 1994; Muñoz et al., 1997; Ćilerdžić et al., 2017). However, the number and activity of secreted isoforms are not only genetically defined, but also depend on substrate type and composition.

Wheat straw and corn stalks are the main lignocellulosics bioconverted to ethanol in Europe and North America, respectively. However, numerous other agro-forestry residues remain unexploited and present serious environmental burdens in numerous regions of the world (Ghaffar et al., 2015). Thus, in many agricultural countries, enormous amounts of cuttings from fruit production remain as environmental ballast. According to data of the Food and Agricultural Organization from 2017, apple and plum were cultures with high annual yields in the European Union (10,106,442 and 1,292,856 metric tons (t), respectively), while Italy and France were the main grape producers, with 7,169,745 and 5,915,882 t, respectively. Serbia was the leader in blackberry production in Europe (27,558 t), the fourth leading world producer of raspberry (68,500 t) and a considerable producer of apple (378,644 t), plum (330,582 t) and grape (165,568 t) in 2013. The mentioned fruit quantities are good indicators of the amount of biomass whose main part remains unused. Additionally, some of them have not yet been studied as substrates for fungal cultivation and raw materials for many industries.

Based on the presented facts, the goals of the study were to characterize the profiles of ligninolytic enzymes in five *Pleurotus* species and determine their ability to delignify eight common but unexploited agro-forestry residues.

## Materials and Methods

### Organisms and Growth Conditions

Cultures of the studied *Pleurotus* spp. were obtained from the Institute of Evolution, University of Haifa, Israel (HAI) and are maintained on malt agar medium in the culture collection of the Institute of Botany, Faculty of Biology, University of Belgrade. The cultures were obtained from fruiting bodies collected in different world regions (Table 1).

**TABLE 1:** Studied *Pleurotus* species.

Species	Code	Origin
*Pleurotus eryngii*	HAI 193	Ukraine, Kherson Region, Chaplinka district, Askania-Nova on *Stipa* sp
*Pleurotus florida*	HAI 217	United States , Florida
*Pleurotus ostreatus*	HAI 1105	Israel, Golan Heights, Massada, on *Quercus calliprinos*
*Pleurotus pulmonarius*	HAI 573	Russia, Sochi
*Pleurotus salignus*	HAI 326	Israel, Dan Natural Reserve, Tel Dan, on *Salix* sp

The inoculum was obtained by inoculation of synthetic medium (glucose, 10.0 g/L; NH_4_NO_3_, 2.0 g/L; K_2_HPO_4_, 1.0 g/L; NaH_2_PO_4_ x H_2_O, 0.4 g/L; MgSO_4_ x 7H_2_O, 0.5 g/L; yeast extract, 2.0 g/L; pH 6.5) with mycelium of 7-day-old culture, grown at room temperature on a rotary shaker for 7 days, washing of harvested biomass with sterile distilled water (dH_2_O) and its homogenization with dH_2_O in a laboratory blender (Stajić et al., 2010).

Solid-state cultivation was carried out during 21 days at 25°C, in the dark, in 250 ml flasks containing 6.0 g of tested plant residues (wheat straw, corn stalks and sawdust of oak, grapevine, blackberry, raspberry, plum and apple) as the carbon source and 30.0 ml of modified synthetic medium (without glucose) to reach a relative humidity of approximately 83%andinoculated with 3.0 ml of prepared inoculum.

The control samples contained medium that was not inoculated but was treated in the same way as the inoculated samples and were used as a negative control for substrate depolymerization.

### Assays of Enzyme Activity and Total Protein Production

Samples were harvested after 21 days of cultivation, and extracellular enzymes were extracted by stirring samples with 50.0 ml dH_2_O on a magnetic stirrer at 4°C for 10 min. The extracts were centrifuged (at 4°C and 3000 rpm for 15 min), and the resulting supernatants were used for spectrophotometric (BioQuest CECIL CE2501, United Kingdom) determination of the activities of Mn-oxidizing peroxidases [Mn-dependent peroxidase (MnP, EC 1.11.1.13) and versatile peroxidase (VP, EC 1.11.1.16)] and laccase (EC 1.10.3.2), as well as total protein content.

The activities of Mn-oxidizing peroxidases and laccases were determined according to the methods of Ćilerdžić et al. (2016) using 3 mm phenol red (ε_610_ = 22,000 M^−1^ cm^−1^) and 2,2^'^-azino-bis-[3-ethyltiazoline-6-sulfonate] (ABTS) (ε_436_ = 29,300 M^−1^ cm^−1^), respectively, as the highly specific substrates for these enzymes. Enzymatic activity was expressed in U/L, where activity of 1U representing the amount of enzyme that transforms 1 μmol of substrate per min.

Total protein content was determined according to Silva et al. (2005) using Bradford’s reagent and bovine serum albumin as the standard and expressed in mg/ml. The obtained value was used to define the specific enzyme activity (U/mg).

### Electrophoresis

Laccase isoforms of the studied *Pleurotus* species cultivated on tested substrates and their isoelectric points (pIs) were determined by isoelectric focusing (IEF) using a Mini IEF Cell 111 (Bio-Rad, United States). Isoelectric focusing was carried out in 7.5% polyacrylamide gel with 5.0% ampholyte on a pH gradient from 3 to 10. Bands were visualized after gel exposure to an ABTS/phosphate buffer (pH 5.0) mixture at room temperature. After completion of focusing, the gel was fixed in trichloroacetic acid, and protein bands were detected by staining with Coomassie Brilliant Blue (CBB) (Stajić et al., 2010). An IEF marker with a pI ranged from 3.6 to 9.3 (Sigma-Aldrich, United States ) was used.

### Defining the Amount of Lignocellulosic Polymers

The loss of substrate dry matter (%) was determined by the formula.

(M*i*–M*f*)/M*i* x 100, where M*i* represents the initial lignocellulosic mass and M*f* is the mass after fermentation by the studied species.

Contents of hemicellulose, cellulose and lignin were measured by modified versions of the methods of Kirk and Obst (1988) and Van Soest et al. (1991). The samples were dried at a 60°C to the constant mass, ground and treated with neutral detergent/Na_2_SO_3_ mixture under refluxing conditions to remove soluble sugars, proteins, lipids and vitamins. The obtained biomass represented neutral detergent fibers (NDF). Acidic detergent fibers (ADF) were obtained by treating the obtained samples with an acidic detergent solution, and the difference between NDF and ADF represented the amount of hemicellulose. Lignin content (LC) was defined after incubation of the samples with 72% H_2_SO_4_ at 30°C and its hydrolysis at 120°C and expressed as percent of the quantity present in the initial sample. Cellulose content was calculated as the difference between ADF and LC.

The efficiency of lignin degradation was expressed as the cellulose enrichment, which represents the ratio between the remaining amounts of cellulose and lignin in treated samples.

### Statistical Analyses

All experiments were done in three replicates and the results were expressed as mean ± standard error. Assaying significant differences between means was performed by one-way analysis of variance (ANOVA) and Tukey’s test, using STATISTICA, version 6.0 (StatSoft, Inc., Tulsa, United States). Statistical significance was declared at *p* < 0.05.

## Results

### Characterization of Ligninolytic Enzymes of *Pleurotus* spp.

**FIGURE 1:**
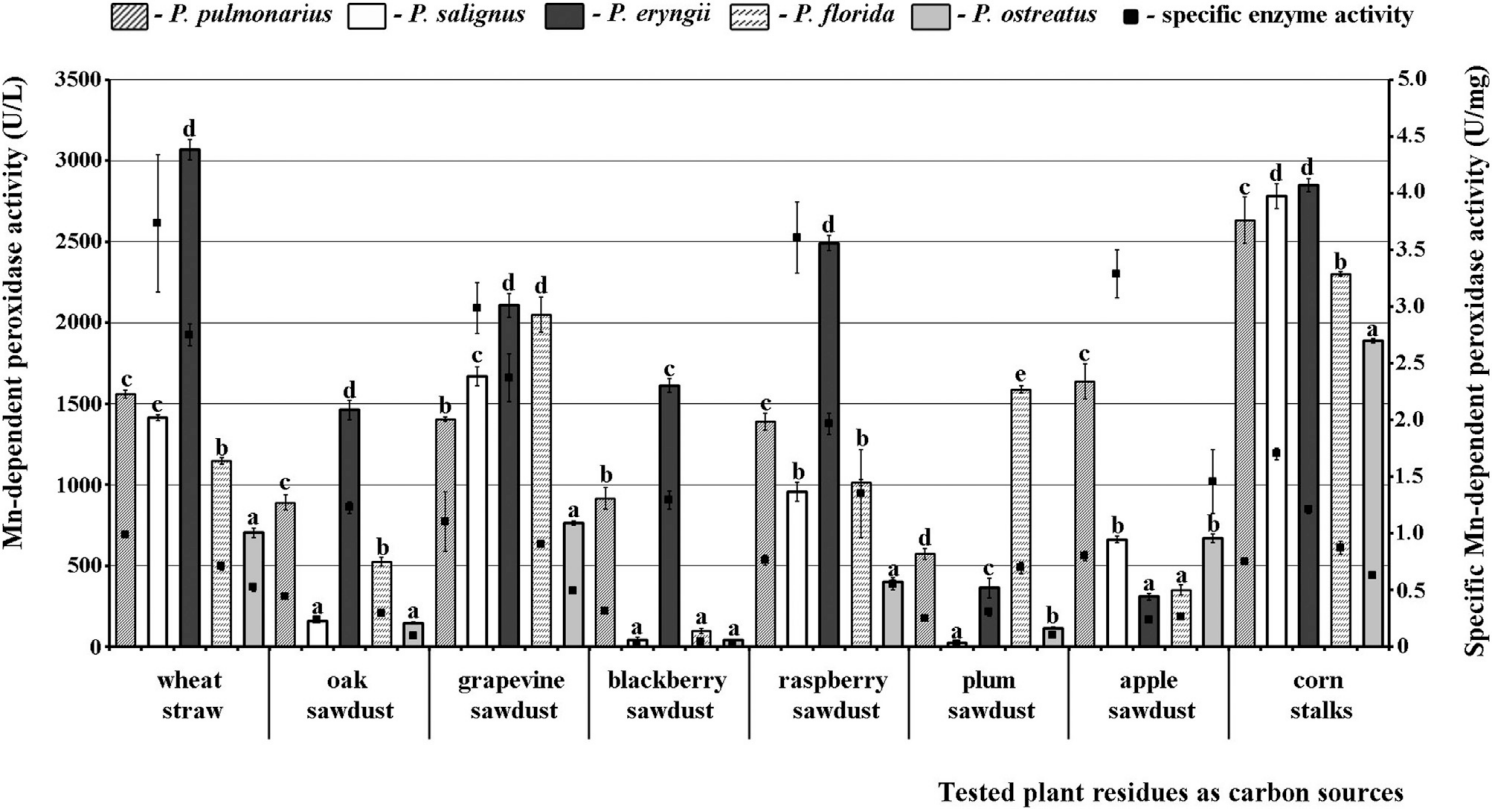
Activity of Mn-dependent peroxidases of *Pleurotus* spp. depending on the type of agro-forestry residues. Values superscripted with the same letter in each values group (for each plant residue) are not significantly different (*p* < 0.05).

The obtained results confirmed that Mn-oxidizing peroxidases and lacasse activities depend on the species of mushroom and type of substrate. To be specific, the studied *Pleurotus* species differed significantly among themselves in their potential for secretion of enzymes on the same substrate, but also each species showed considerable variability of enzymes activities on different substrates. Thus, *P*. *eryngii* HAI 193 was the best producer of highly active forms of MnP on most substrates except plum and apple sawdusts, while *P*. *ostreatus* HAI 1105 and *P*. *salignus* HAI 326 were the weakest MnP producers on almost all residues (Figure 1).

As for the effect of substrate type, maximal MnP activity in *P. eryngii* was measured after fermentation of wheat straw (3066.92 U/L), while this activity was slightly lower on corn stalks (2848.48 U/L) and lowest on apple sawdust (308.71 U/L). However, although the highest MnP activity was noted on wheat straw, corn stalks were the optimal inducer of enzyme production in all of the other studied species, i.e., this activity in them ranged between 1888.89 U/L and 2848.48 U/L (Figure 1).

**FIGURE 2:**
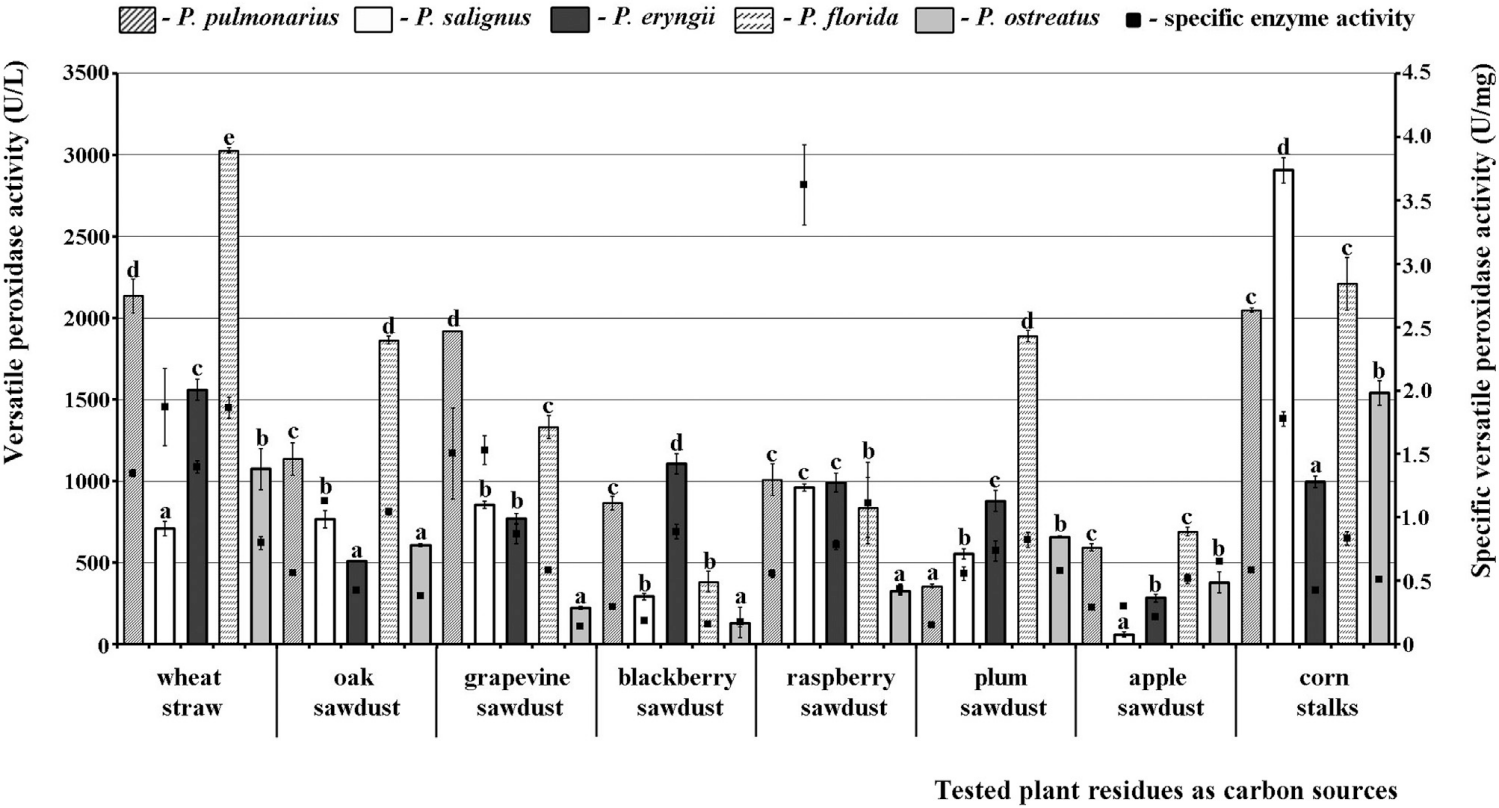
Activity of versatile peroxidases of *Pleurotus* spp. depending on the type of agro-forestry residues. Values superscripted with the same letter in each values group (for each plant residue) are not significantly different (*p* < 0.05).

A different picture was observed for VP. Thus, *P*. *florida* HAI 217 produced highly active VP isoforms on almost all of the tested substrates with the maximal activity on wheat straw, while the lowest activity was noted in *P. salignus* HAI 326 on apple sawdust. Wheat straw and corn stalks stood out as the best substrates for production of highly active VP isoforms, with peaks of 3028.41 U/L and 2905.30 U/L noted in *P. florida* and *P. salignus*, respectively (Figure 2). On the other hand, apple and blackberry sawdusts were the weakest stimulators of VP activity (60.61 U/L in *P. salignus* and 128.79 U/L in *P. ostreatus*, respectively).

**FIGURE 3:**
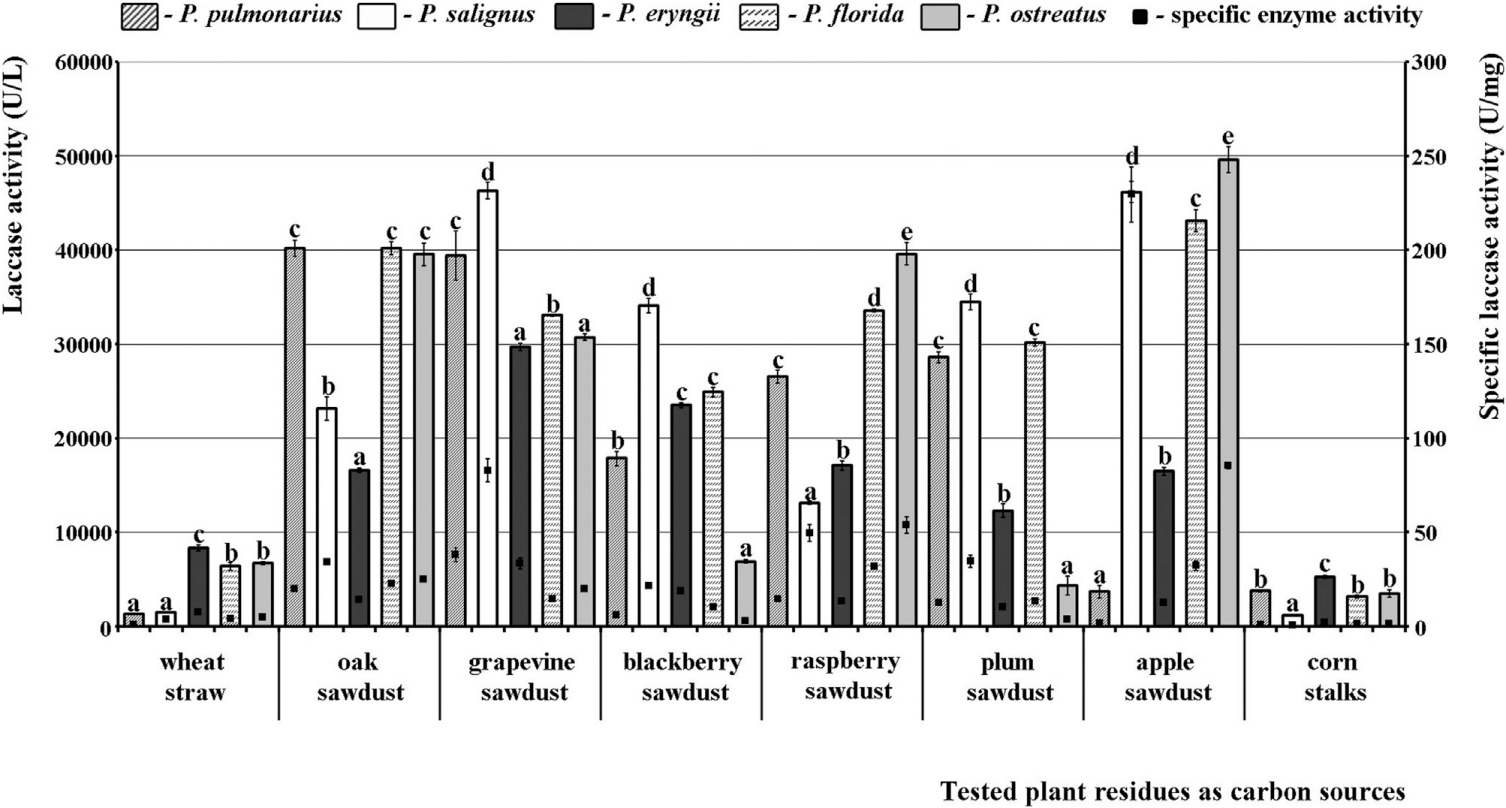
Activity of laccases of *Pleurotus* spp. depending on the type of agro-forestry residues. Values superscripted with the same letter in each values group (for each plant residue) are not significantly different (*p* < 0.05).

In the case of laccase, even three species were good producers, *P*. *florida*, *P*. *salignus*, and especially *P. ostreatus*, in which the highest activity was noted. As for the effect of the studied substrates on the activity profile, it was absolutely contrary to the situation with MnP and VP. To be specific, the tested sawdusts were good stimulators, while wheat straw and corn stalks did not induce the synthesis of highly active laccase isoforms. Thus, the highest activity was obtained in apple sawdust fermentation by *P. ostreatus* (49601.82 U/L), although grapevine, oak and raspberry sawdusts also induced laccase activity of about 40000.00 U/L in many species. The lowest activity was observed after fermentation of corn stalks by *P. salignus* (1160.42 U/L), but it was also low after fermentation of wheat straw by *P. pulmonarius* and *P. salignus* (1262.80 U/L and 1433.45 U/L, respectively) (Figure 3).

Regarding the purity of the enzymes, the highest specific activities of MnP (3.73 U/mg), VP (3.62 U/mg) and laccase (229.50 U/mg) were noted in *P*. *salignus* cultivated on wheat straw, raspberry and apple sawdust, respectively (Figures 1–3).

**FIGURE 4:**
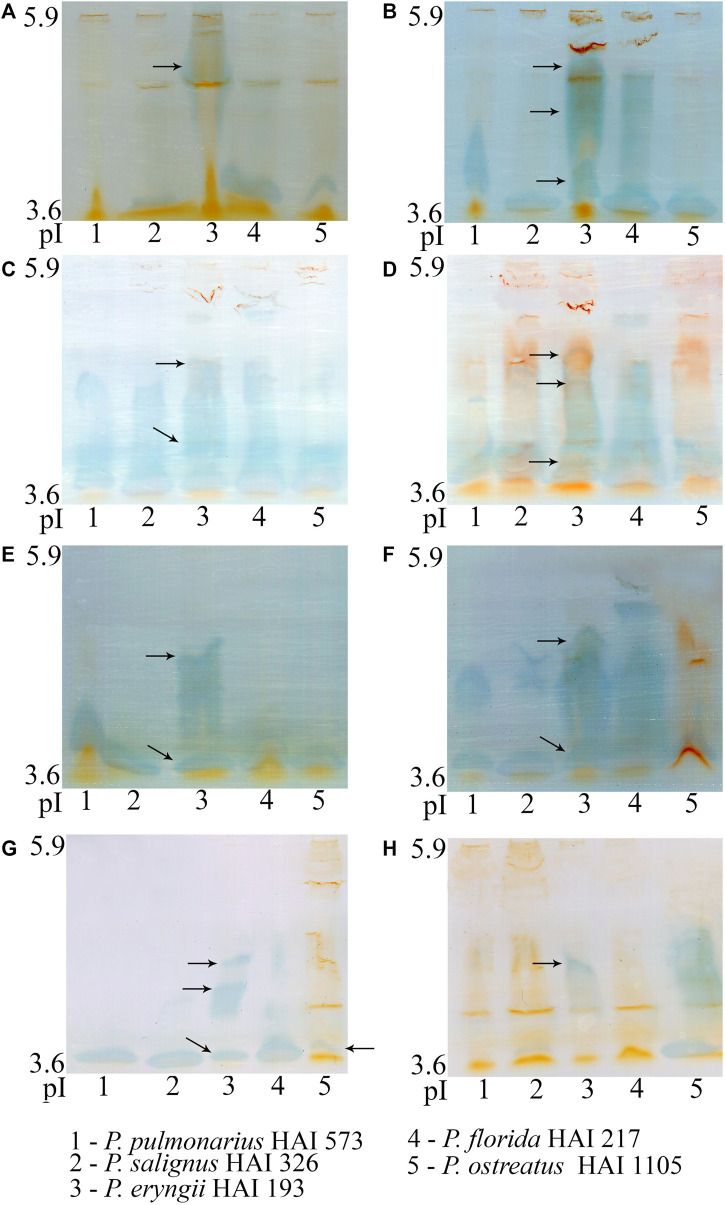
Isoelectric focusing pattern of *Pleurotus* spp. laccases after 21 days of solid-state fermentation of wheat straw **(A)**, oak sawdust **(B)**, grapevine sawdust **(C)**, blackberry sawdust **(D)**, raspberry sawdust **(E)**, plum sawdust **(F)**, apple sawdust **(G)** and corn stalks **(H)**.

Because it showed the highest production and activity, laccase was chosen for further characterization. The number, intensity and pI of visualized laccase isoforms on the same substrate varied among the studied species, but also differed for each species on various substrates (Figure 4). Thus, oak, grapevine, blackberry, raspberry and plum sawdusts induced synthesis of more numerous or/and more intensive isoforms in all of the studied species. However, it should be emphasized that the number and intensity of isoforms did not coincide with the measured activity in all species and on all substrates. Although *P*. *ostreatus* produced the most active laccase during apple sawdust fermentation, only one isoenzyme with pI about 3.6 was visualized (Figure 4). On the other hand, in *P. eryngii,* whose laccase activity was moderate, several isoforms with pIs of about 3.6, 4.6 and 5.3 were visualized on all substrates except corn stalks, where *P. ostreatus* was dominant with respect to the number of isoforms, despite very low activity (Figure 4).

### Degradation of Lignocellulosic Biomass

Generally, the percentage of dry matter loss was substrate- and species-dependent. Thus, the tested species showed significant differences in the capacity for reduction of the total dry mass of tested substrates (Table 2). The greatest loss of dry matter was noted for wheat straw, where the highest level of reduction (24.83%) was obtained in the case of *P*. *florida*. On the other hand, plum and oak sawdusts were the most resistant to the effect of *Pleurotus* spp. enzymes, and their dry matter losses were the lowest (8.83 and 9.67%, respectively, by *P. pulmonarius*). However, a low correlation between dry mass loss and MnP, VP and laccase activities was noted (R^2^ = 0.06, 0.18 and 0.09, respectively).

**TABLE 2:** Extent of depolymerization of plant residues by *Pleurotus* spp.

Plant residue	Studied samples	Sample weight (g)	Fibres composition of samples (mg)			Dry matter loss (%)	Extent of polymers degradation (%)			Cellulose enrichment
			Lignin	Cellulose	Hemicellulose		Lignin	Cellulose	Hemicellulose	
**Wheat straw**	Control*	6.00	666.00	2418.00	1692.00	—	—	—	—	—
	*P. pulmonarius*	4.77	319.86	2091.01	1050.28	20.50^d^	51.97^d^	13.52^c^	37.93^d^	6.54^c^
	*P. salignus*	4.88	434.14	2258.51	1009.75	18.67^b^	34.81^c^	6.60^a^	40.32^e^	5.20^b^
	*P. eryngii*	5.16	443.85	2152.14	1269.61	14.00^a^	33.36^b^	11.00^b^	24.96^a^	4.85^b^
	*P. florida*	4.51	446.79	1769.10	1119.22	24.83^e^	32.91^b^	26.84^e^	33.85^c^	3.96^a^
	*P. ostreatus*	4.81	499.82	1965.65	1158.25	19.83^c^	24.95^a^	18.71^d^	31.55^b^	3.93^a^
**Oak sawdust**	Control*	6.00	1530.00	2808.84	1159.97	—	—	—	—	—
	*P. pulmonarius*	5.42	1040.45	2454.81	1013.35	9.67^a^	32.00^d^	12.60^c^	12.60^b^	2.36^b^
	*P. salignus*	5.31	1168.86	2629.94	892.58	11.50^c^	23.60^c^	6.37^a^	23.05^d^	2.25^b^
	*P. eryngii*	5.38	1237.40	2383.34	1097.52	10.33^b^	19.12^b^	15.15^d^	5.38^a^	1.93^a^
	*P. florida*	5.34	1174.14	2593.78	1008.69	11.00^c^	23.26^c^	7.66^b^	13.04^c^	2.21^b^
	*P. ostreatus*	5.36	1253.30	2485.18	1017.64	10.67^b^	18.08^a^	11.52^c^	12.27^b^	1.98^a^
**Grapevine sawdust**	Control*	6.00	1421.41	2652.00	887.08	—	—	—	—	—
	*P. pulmonarius*	4.93	1134.36	1898.82	695.41	17.83^c^	20.19^e^	28.40^c^	21.61^c^	1.67^a^
	*P. salignus*	5.13	1283.25	2160.99	600.56	14.50^b^	9.72^c^	18.51^a^	32.30^e^	1.68^a^
	*P. eryngii*	5.29	1201.74	2170.54	794.10	11.83^a^	15.45^d^	18.15^a^	10.48^a^	1.81^b^
	*P. florida*	5.15	1333.07	2177.18	756.61	14.17^b^	6.21^b^	17.90^a^	14.71^b^	1.63^a^
	*P. ostreatus*	5.11	1362.00	2116.78	654.46	14.83^b^	4.18^a^	20.18^b^	26.22^d^	1.55^a^
**Blackberry sawdust**	Control*	6.00	1218.00	2712.00	1038.00	—	—	—	—	—
	*P. pulmonarius*	4.81	726.16	2183.29	706.92	19.83^c^	40.38^d^	19.50^b^	31.90^c^	3.01^c^
	*P. salignus*	4.86	850.68	1968.71	709.71	19.00^c^	30.16^c^	27.41^d^	31.63^c^	2.31^b^
	*P. eryngii*	5.12	834.23	2267.27	946.83	14.67^a^	31.51^c^	16.40^a^	8.78^a^	2.72^c^
	*P. florida*	5.06	895.27	2169.88	723.29	15.67^b^	26.50^b^	19.99^b^	30.32^b^	2.42^b^
	*P. ostreatus*	5.02	988.15	2081.64	707.26	16.33^b^	18.87^a^	23.24^c^	31.86^c^	2.04^a^
**Raspberry sawdust**	Control*	6.00	1200.00	2160.00	1308.00	—	—	—	—	—
	*P. pulmonarius*	4.84	885.35	1940.04	619.26	19.33^c^	26.22^e^	10.18^a^	52.66^e^	2.19^b^
	*P. salignus*	4.94	934.23	1823.97	726.62	17.67^b^	22.15^c^	15.56^b^	44.45^b^	1.95^a^
	*P. eryngii*	5.02	903.06	1796.09	988.35	16.33^a^	24.75^d^	16.85^c^	24.44^a^	1.99^a^
	*P. florida*	4.84	1026.93	1932.76	678.16	19.33^c^	14.42^b^	10.52^a^	48.15^c^	1.88^a^
	*P. ostreatus*	4.99	1057.24	1924.98	658.28	16.83^a^	11.90^a^	10.88^a^	49.67^d^	1.82^a^
**Plum sawdust**	Control*	6.00	1837.49	2544.00	1368.00	—	—	—	—	—
	*P. pulmonarius*	5.47	1509.44	2029.00	738.32	8.83^a^	17.85^d^	20.24^b^	46.03^b^	1.34^b^
	*P. salignus*	5.27	1729.54	1919.37	680.22	12.17^c^	5.87^a^	24.55^c^	50.28^c^	1.11^a^
	*P. eryngii*	5.34	1569.37	1868.30	848.74	11.00^b^	14.59^c^	26.56^d^	37.96^a^	1.19^a^
	*P. florida*	5.22	1696.18	2155.45	594.97	13.00^d^	7.69^b^	15.27^a^	56.51^d^	1.27^b^
	*P. ostreatus*	5.31	1719.79	1868.42	721.89	11.50^b^	6.41^a^	26.56^d^	47.23^b^	1.09^a^
**Apple sawdust**	Control*	6.00	1158.00	2808.00	1176.00	—	—	—	—	—
	*P. pulmonarius*	5.01	881.58	2068.72	931.69	16.50^b^	23.87^d^	26.33^c^	20.78^b^	2.35^b^
	*P. salignus*	4.80	1003.41	2290.08	691.34	20.00^e^	13.35^c^	18.44^b^	41.21^d^	2.28^a^
	*P. eryngii*	5.16	862.22	2070.36	1048.09	14.00^a^	25.54^e^	26.27^c^	10.88^a^	2.40^b^
	*P. florida*	4.85	1022.93	2419.15	814.46	19.17^d^	11.66^b^	13.85^a^	30.74^c^	2.36^b^
	*P. ostreatus*	4.95	1043.82	2290.46	638.16	17.50^c^	9.86^a^	18.43^b^	45.73^e^	2.19^a^
**Corn stalks**	Control*	6.00	594.00	2796.00	1860.43	—	—	—	—	—
	*P. pulmonarius*	4.93	409.52	2452.20	1036.14	17.83^b^	31.06^b^	12.30^a^	44.31^c^	5.99^b^
	*P. salignus*	4.64	338.50	1961.45	1029.41	22.67^d^	43.01^e^	29.85^c^	44.67^c^	5.79^b^
	*P. eryngii*	5.16	366.57	2060.04	1270.10	14.00^a^	38.29^d^	26.32^b^	31.73^a^	5.62^b^
	*P. florida*	4.66	381.96	1872.52	1043.39	22.33^d^	35.70^c^	33.03^d^	43.92^c^	4.90^a^
	*P. ostreatus*	4.76	423.91	1948.07	1105.02	20.67^c^	28.64^a^	30.33^c^	40.60^b^	4.60^a^

*Uninoculated plant residue.

^a–e^Values superscripted with the same letter in the same sub-column (for each plant residue) are not significantly different (*p* < 0.05).

The tested species varied with respect to the degree of lignin and cellulose degradation (Table 2). Thus, *P*. *pulmonarius* was the most effective degrader of wheat straw lignin (51.97%) and slightly weaker mineralizator of blackberry sawdust lignin (40.38%). Another member of the group of good degraders is *P*. *salignus*, which caused a 43.01%. reduction of lignin content in corn stalks-based substrate. On the other hand, grapevine sawdust was the most resistant to the studied ligninosomes, especially to *P. ostreatus* one, which degraded only 4.18% of this polymer. However, although *P. pulmonarius* and *P. salignus* were the best delignificators of the mentioned residues, the activities of their ligninolytic enzymes were not the highest at a given measurement point, which is confirmed by the correlation coefficients between the amount of decomposed lignin and the activity of MnP, VP and laccase (R^2^ = 0.18, 0.26, 0.37, respectively), which could be explained by the dynamics of the synthesis of highly active forms of these enzymes.

Although the main aim of this study was to define the cultivation conditions for maximal lignin removal and cellulose preservation, it was found that the studied species degraded cellulose to a certain extent, depending on the substrate type. *Pleurotus florida*, *P. ostreatus* and *P. salignus* caused the maximal loss of cellulose in corn stalks (33.03, 30.33 and 29.85%, respectively), in contrast to oak sawdust, where *P. salignus* mineralized only 6.37% of cellulose (Table 2).

However, the degree of lignin and cellulose degradation is not a unique indicator of species and substrate applicability in various biotechnological processes. The main indicator is degradation selectivity, expressed as the ratio between remaining cellulose and lignin amounts, i.e., cellulose enrichment. Thus, the highest cellulose enrichment was noted in *P*. *pulmonarius* (6.54) which was the most effective degrader of wheat straw lignin but the weak mineralizator of its cellulose. On the other hand, *P. ostreatus* was the weakest delignifier of grapevine sawdust (4.18%), but good consumer of its cellulose which induced the low cellulose enrichment (1.55) (Table 2).

## Discussion

Lignocellulose-based biomass is an abundant and renewable resource for the production of bioethanol, paper, feed, food and numerous value-added products (Stajić et al., 2009; Rastogi and Shrivastava, 2017). Well-known shortcomings of conventional physico-chemical delignification methods have led to a growing need to find a more efficient biological pretreatment of lignocellulose in order to realize sucessful utilization. That has encouraged a huge number of studies examining fungi as the most prominent delignifiers. Although species of the genus *Pleurotus* are known as effective lignocellulosic depolymerizers, the majority of recent studies have addressed the ligninolytic potential of *P. ostreatus* with scarce data about the other species of this genus (Stajić et al., 2004, 2006; Singh and Singh, 2014; Aditiya et al., 2016; Bilal et al., 2017; Alfaro et al., 2020). Compared to previous reports, the *Pleurotus* spp. profiled in the present research showed an encouraging ligninolytic capacity in relation to various agro-forestry residues. The tremendous influence of the lignocellulosic substrate type on laccase activity has been already shown for *Pleurotus* spp. by Stajić et al. (2006). However, in comparison with our results, those authors reported many times lower laccase activity for *P. eryngii*, *P. ostreatus* and *P. pulmonarius* strains after 7 days of fermentation of tangerine peels and grapevine sawdust. Significantly lower laccase activity (∼500 U/L) was also observed after 3 weeks of sugarcane bagasse fermentation by the *P. ostreatus* strain studied by Dong et al. (2013), a value of about 35000 U/L being achieved only after 6 weeks of cultivation. Previous studies also reported an effect of substrate type on the number of visualized laccase isoforms. Thus, Muñoz et al. (1997) obtained two laccase bands after fermentation of glucose/ammonium-tartrate medium by *P. ostreatus*, Palmieri et al. (1997) obtained four bands after submerged cultivation in potato dextrose/yeast extract medium, while Stajić et al. (2006) observed expression of three isoforms on grapevine sawdust. However, there are numerous data that the activity of enzymes is not always positively correlated with the number of visualized isoforms, i.e., it often happens that the mushroom synthesizes a larger number of isoforms of lower activities or only a few or one isoform of high activity.

On the other hand, Dong et al. (2013) reported an extremely high activity of MnP, even 150000 U/L, after 3 weeks of sugarcane bagasse fermentation by *P. ostreatus.* This enzyme could be responsible for the recorded significant loss of total dry mass (8%) and lignin degradation (47%). A similar extent of delignification was also caused by the *P. ostreatus* strain studied by Asgher et al. (2014) cultivated on corn stalks and wheat straw, when 54 and 45.7% of lignin, respectively, was removed. In our study, *P. pulmonarius* and *P. salignus* caused a similar loss of dry biomass and lignin from wheat straw and corn stalks, respectively, despite low laccase activity. The high level of lignin depolymerization can be attributed to highly active Mn-oxidizing peroxidases, which have a crucial role in the initial phase of the process when a large amount of generated Mn^3+^ makes up for the decreased action of laccases due to their limited diffusion into the substrate as a result of their oversized molecules (Knežević et al., 2016; Stajić et al., 2016). A level of rice straw delignification (25%) similar to that obtained for the as strain tested in our study during cultivation on wheat straw was caused by a Chinese strain of *P. ostreatus* (Mustafa et al., 2016). However, a considerably lower delignification potential was recorded in *P. sajor-caju*, used for the pretreatment of residues collected at a co-digestion biogas plant, where only 8.7% of lignin was degraded after 6 weeks of cultivation (Fang et al., 2018). Another weak delignifier was *P. eryngii* as studied by Martínez-Patiño et al. (2018), which caused only 1.5% lignin mineralization after even 45 days of fermentation of olive tree leaves owing to extremely low MnP and laccase activities (8.0 U/g and 3.0 U/g, respectively).

Similar to the high percentage of plant raw materials delignification, a high level of its hemicellulose degradation was measured in our study, which Martínez et al. (1994) explained by the preference of white rot fungi to decompose lignin and xylan over the other polymers. Thus, even 47% of lignin and 43% of xylan loss was detected after *P. eryngii* cultivation on wheat straw which only a 14% of cellulose was degraded (Camarero et al., 1994).

The origin of strains and their genetic predisposition certainly have a very important effect on their delignification selectivity. Thus, in contrast to the *P. florida* strain tested in the present study, which showed a low cellulose enrichment, the strains studied by Rouches et al. (2016) caused highly selective delignification of wheat straw and corn stalks, successfully pretreating them for biogas production. Mustafa et al. (2016) also reported a high selectivity for a Chinese *P. ostreatus* strain during rice straw fermentation. On the other hand, extremely low selectivity was noted after fermentation of banana leaf-based waste by an Indian *P. florida* strain, which caused about 30% lignin removal and even 50–70% cellulose removal (Chanakya et al., 2015).

Generally, the obtained results clearly singled out *P. pulmonarius* HAI 573 as highly efficient and selective delignifier, especially of wheat straw, which opens new possibilities for the use of its ligninolytic cocktail in pretreatment of this raw material, as the first phase in numerous biotechnological processes, primarily in bioethanol and paper production.

## Conclusion

The main contribution of the present study is the characterization of the ligninolytic enzyme system and lignocellulose degradation capacity of insufficiently studied *Pleurotus* species. Most of the substrates considered in the study were tested for the first time in despite their great abundance and demonstrated high potential for induction of strong ligninolytic enzymes production by *Pleurotus* spp. This opens up a huge space for future detailed studies aiming to include these lignocellulosics in a number of biotechnological processes. Pretreated with the most selective degraders, those lignocellulosics could be used for the production of more digestable feed, paper, bioethanol and other valued compounds. The present study has clearly confirmed that naturally destructive organisms such as *Pleurotus* spp., i.e., a cocktail of their ligninolytic enzymes, could be potentially employed in very useful biotechnological processes making possible the utilization of common lignocellulosic wastes. The obtained results can serve as the basis for further optimization of high-scale production of the most active enzyme isoforms for industrial utilization.

## Data Availability

The original contributions presented in the study are included in the article/Supplementary Material, further inquiries can be directed to the corresponding author.
